# Dopaminergic Control of the Globus Pallidus through Activation of D2 Receptors and Its Impact on the Electrical Activity of Subthalamic Nucleus and Substantia Nigra Reticulata Neurons

**DOI:** 10.1371/journal.pone.0119152

**Published:** 2015-03-05

**Authors:** Omar Mamad, Claire Delaville, Wail Benjelloun, Abdelhamid Benazzouz

**Affiliations:** 1 Univ. de Bordeaux, Institut des Maladies Neurodégénératives, UMR 5293, F-33000, Bordeaux, France; 2 CNRS, Institut des Maladies Neurodégénératives, UMR 5293, F-33000, Bordeaux, France; 3 Université Mohamed V-Agdal, Faculté des Sciences, Equipe Rythmes Biologiques, Neurosciences et Environnement, 10000, Rabat, Morocco; Florey Institute of Neuroscience and Mental Health, The University of Melbourne, AUSTRALIA

## Abstract

The globus pallidus (GP) receives dopaminergic afferents from the *pars compacta* of substantia nigra and several studies suggested that dopamine exerts its action in the GP through presynaptic D2 receptors (D2Rs). However, the impact of dopamine in GP on the pallido-subthalamic and pallido-nigral neurotransmission is not known. Here, we investigated the role of dopamine, through activation of D2Rs, in the modulation of GP neuronal activity and its impact on the electrical activity of subthalamic nucleus (STN) and substantia nigra *reticulata* (SNr) neurons. Extracellular recordings combined with local intracerebral microinjection of drugs were done in male Sprague-Dawley rats under urethane anesthesia. We showed that dopamine, when injected locally, increased the firing rate of the majority of neurons in the GP. This increase of the firing rate was mimicked by quinpirole, a D2R agonist, and prevented by sulpiride, a D2R antagonist. In parallel, the injection of dopamine, as well as quinpirole, in the GP reduced the firing rate of majority of STN and SNr neurons. However, neither dopamine nor quinpirole changed the tonic discharge pattern of GP, STN and SNr neurons. Our results are the first to demonstrate that dopamine through activation of D2Rs located in the GP plays an important role in the modulation of GP-STN and GP-SNr neurotransmission and consequently controls STN and SNr neuronal firing. Moreover, we provide evidence that dopamine modulate the firing rate but not the pattern of GP neurons, which in turn control the firing rate, but not the pattern of STN and SNr neurons.

## Introduction

The globus pallidus (GP, the rodent homologue of the primate globus pallidus externus, GPe) is a basal ganglia structure playing a key role in the control of movement. It is considered as an inhibitory GABAergic relay in the indirect pathway, linking the striatum to the *pars reticulata* of substantia nigra (SNr), directly or indirectly via the subthalamic nucleus (STN) [1]. Major GP afferents originate from the striatum and use GABA as a neurotransmitter, while its glutamatergic afferents arise from the STN [2] and the parafascicular nucleus of the thalamus [3]. In addition, GP receives dopaminergic projections from the *pars compacta* of substantia nigra (SNc) [4]. While the striatum is by far the main target of SNc dopamine neurons, dopamine also mediates its regulatory function at the level of GP [5,6]. Both dopamine D1 (D1R) and D2 (D2R) receptor families are expressed in the GP with a predominance of D2Rs [7]. Most of the presynaptic dopamine receptors are thought to be D2Rs, and are located on terminals of the GABAergic and glutamatergic afferents with lower levels of D1Rs detected in axons and terminal buttons in GP [6–8]. From a functional point of view, a major role of dopamine in the modulation of GP neuronal activity has been suggested by studies demonstrating that intrapallidal dopamine receptor blockade [9] or dopamine depletion [10] produced motor deficits in rodents that can be associated with a reduction of the firing rate of GP neurons [11,12].

Given the predominance of D2Rs in GP and that most actions of dopamine in the GP are mediated by D2Rs [5], we investigated the role of dopamine, through activation of D2Rs, in the control of GP neuronal activity using *in vivo* extracellular recordings in the rat. Then, as the GP is tightly interconnected with the STN and SNr, we studied the impact of dopamine in the GP on the control of pallido-subthalamic and pallido-nigral neurotransmission.

## Materials and Methods

### Ethics statement

All animal experiments were performed in accordance with European Communities Council Directive 2010/63/UE. The study received approval from the local Ethics Committee under the number 50120136-A (Comité d’éthique pour l’expérimentation animale Bordeaux, France). All efforts were made to minimize the number of animals used and their suffering.

### Animals

Adult male Sprague Dawley rats, weighing 280–380 g, were used for *in vivo* electrophysiological experiments under anesthesia. Animals were provided by the “Centre d’Elevage Depré” (Saint Doulchard, France) and arrived at least 1 week before use. They were housed four per cage under artificial conditions of light (12/12 light/dark cycle; lights on at 7:00 A.M.), temperature (24°C), and humidity (45%) with food and water available *ad libitum*.

### Drugs

Drugs were chosen on the basis of affinity for their preferential receptors. Dopamine, purchased from Sigma (Saint-Quentin Fallavier, France), was used at the dose of 2 μg dissolved in 200 nl of 0.9% NaCl. Quinpirole (Sigma) was chosen as a D2R agonist and three doses were tested (0.2, 0.4 and 0.8 μg) to investigate the dose-response on GP neuronal activity. These doses were also dissolved in 200 nl of 0.9% NaCl. Concerning the experiments with the injection and recording in the GP, a small volume of 20 nl of each solution was used to avoid the risk of losing the recorded cell due to the local pressure injection. When the injection was done in GP and recordings in the STN or SNr, a volume of 200 nl was used to have a larger diffusion into the nucleus. 200 nl was selected after a series of control tests, using the pontamine sky blue, in which this volume showed a diffusion of the solution into the GP without a spread outside the nucleus. By using this volume, the injected drug exerts its effect everywhere in the nucleus to affect GP cells projecting to the recorded neurons in the STN and SNr. It is unlikely that this volume may exert different effects compared to 20 nl as the concentration is the same and the proportions of the responsive excited and inhibited STN and SNr neurons were concordant with those of GP neurons (see results). Dopamine D2Rs were blocked by intra-peritoneal injection of sulpiride, a selective D2R antagonist (40 mg/kg), which was dissolved in 2 ml sterile injectable water to which was added HCl and the final pH of 6.5–7.2 was titrated with NaOH. The final pH of dopamine and quinpirole solutions was between 7.0 and 7.2.

### Extracellular recordings and drugs microinjections

Extracellular single-unit recordings were made in rats anesthetized with urethane (1.2 g/kg, i.p.). For recording and simultaneous microinjection of drugs in the GP, a double-barreled pipette assembly, similar to that described previously [13,14], was used. The tips of recording and injection micropipettes were separated by 150 to 200 μm. For recording in the STN or SNr and simultaneous microinjection of drugs in the GP, the injection micropipette was placed in the GP and the recording electrode was placed in the STN or SNr. The injection micropipette was filled either with dopamine or with quinpirole drugs and the recording electrode, with an impedance of 8 to 12 MΩ, was filled with 4% pontamine sky blue in 0.9% NaCl. The micropipettes were placed into the targeted nuclei according to the stereotactic coordinates given in the brain atlas of Paxinos and Watson [15] for the GP (AP: 0.9 mm posterior to bregma, L: 3 mm from the midline, DV: 5.5–7.5 mm from the dura), STN (AP: -3.8 mm posterior to bregma, L: 2.5 mm from the midline, DV: 7.5–8.5 mm from the dura) and SNr (AP: -5.3 mm posterior to bregma; L: 2.5 mm from the midline; DV: 7.5–8.5 mm from the dura). Extracellular neuronal activity was amplified, bandpass-filtered (300–3000 Hz) using the Neurolog system (Digitimer, Hertfordshire, UK), displayed on an oscilloscope and transferred via a Powerlab interface to a computer equipped with Chart software (AD Instruments, Charlotte, USA). Only neuronal activity with a signal-to-noise ratio of 3:1 was recorded and used for additional investigation. Basal firing of GP, STN and SNr neurons was recorded for 20 min. before drug injection to ascertain the stability of the discharge activity. A dopaminergic drug or the saline vehicle was then injected into the GP at a volume of 20 nl, using brief pulses (200 ms) of pneumatic pressure (Picospritzer III, Royston Herts, UK). In all rats, the central part of the nucleus was targeted. At the end of each session, the recording site was marked by electrophoretic injection (Iso DAM 80, WPI, Hertfordshire, UK) of Pontamine sky blue through the micropipette at a negative current of 20 μA for 8 min. Recording sequences of 10 minutes each were used for off-line data analysis of GP, STN and SNr neuronal activity recorded before and after drug injection. We used a spike discriminator program (spike histogram program, AD Instruments, Charlotte, USA), and firing parameters were determined using Neuroexplorer (Alpha Omega, Nazareth, Israel). After the drug injection, the start of an excitatory effect was considered when the firing rate was higher than the mean+(2xSD) of the baseline and the start of an inhibitory effect was considered when the firing rate was lower than the mean-(2xSD) (SD = standard deviation). The minimum period of time accepted as a significant effect was 10 seconds and the end of an effect was defined when the firing rate returned to the same value relative to baseline. The firing patterns of GP, STN and SNr neurons were analyzed using the coefficient of variation of the interspike intervals as well as the density histograms according to the method developed by Kaneoke and Vitek [16], as previously described [13,17]. An algorithm using Matlab computer software was used allowing the discrimination of tonic regular, irregular and burst firing.

### Validation of the recording sites

After completion of the experiments, animals were sacrificed by an overdose of urethane, the brains were removed, frozen in isopentane at -45°C and stored at -80°C. Fresh-frozen brains were cryostat-cut into 20 μm coronal sections and acetylcholine esterase staining was used as previously described [18] to determine the location of the Pontamine sky blue dots marking the recording site in the recorded structure. Only brains in which both the recording and drug injection were shown to be in the targeted structure were used for data analysis.

### Statistical analysis

Data are presented as mean ± S.E.M. Statistical analyses were performed using Sigmaplot (Systat Software, San Jose, USA). Firing rates and coefficients of variation of interspike intervals during the 20 minutes before and the 20 minutes after drug injection were compared using paired student *t*-test. The effects of DA alone or combined with the injection of sulpiride were compared using One Way ANOVA followed, when significant, by a multiple comparison procedures using student Newman-Keuls test. The distribution of the firing patterns was assessed using a Chi^2^ test.

## Results

### Effects of intrapallidal injection of dopamine and quinpirole on the spontaneous firing of GP neurons

**Fig 1:**
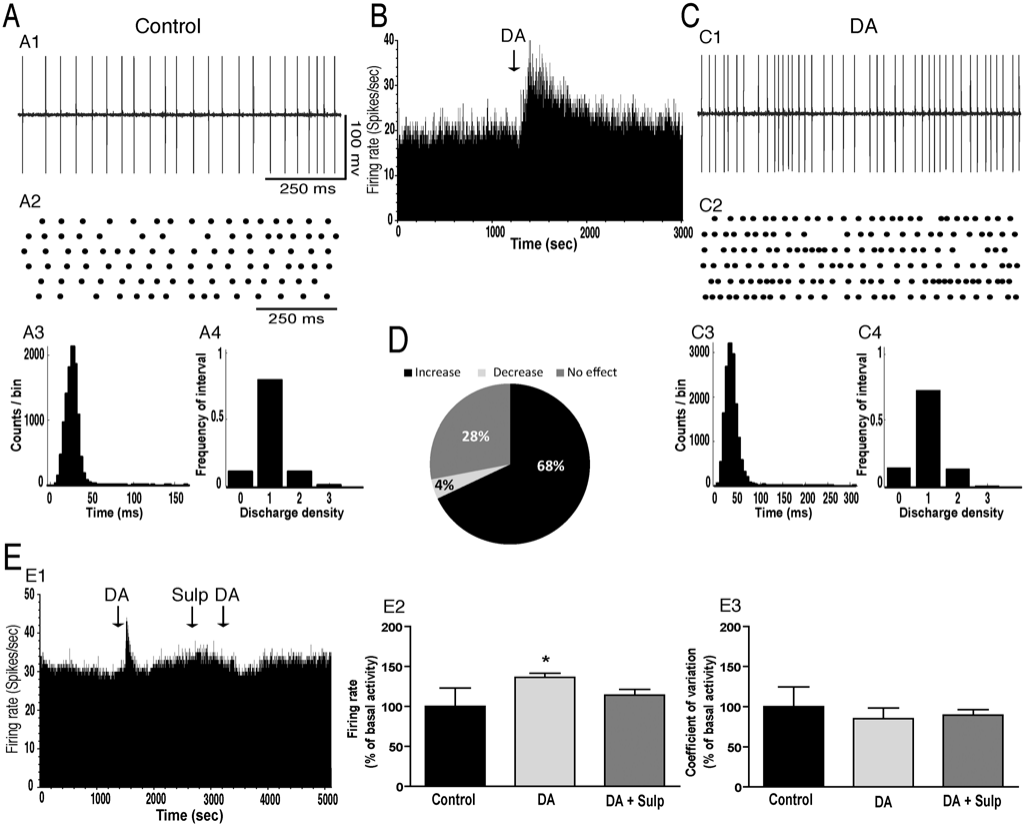
Intrapallidal microinjection of dopamine predominantly increased the firing rate without changing the tonic firing pattern of GP neurons. (A-C) A representative example of GP neuron before (AB) and after (BC) microinjection of dopamine (DA) into the GP showing an increase of its firing rate with spike train (A1C1), firing rate histogram (B), raster display of random segments of recording (A2C2), insterspike interval histogram (A3C3) and density histogram (A4C4) of the same GP neuron. (D) Circular plot representing the percentage of GP neurons showing an increase, a decrease or no change of their firing rate after the local injection of dopamine. (E) Representative firing rate histogram with the response of a GP neuron showing an increase of its firing rate after dopamine injection corresponding to the effect observed in the majority of GP neurons. The excitatory effect of dopamine (DA) was prevented by the selective D2R antagonist, sulpiride (Sulp). Note that DA increased the firing rate (E1E2) without changing the coefficient of variation (E3) of GP neurons and that after the injection of sulpiride (DA+Sulp), DA had no effect on the firing rate (E1E2). *p<0.05

The effects of local injection of dopamine and the D2R agonist, quinpirole, on the spontaneous discharge of GP neurons were investigated in 40 animals under urethane anesthesia. In control conditions, the mean firing rate of GP neurons was 19.32±1.05 spikes/sec (n = 84) and all GP cells exhibited a tonic discharge pattern as shown by the interspike intervals and density histograms (Fig. 1A). Control microinjection of saline (0.9% NaCl) into the GP revealed no significant effects on the firing rate and pattern of neuronal activity in the GP (data not shown). However, intrapallidal injection of dopamine predominantly induced an excitatory effect on GP neurons (Fig. 1BC). It significantly increased the firing rate of 34 out of 50 GP neurons (68%, Fig. 1D) with a percentage increase of 45% (*p*<0.001, paired *t*-test, Fig. 1B–D, Table 1). This effect occurred within 2–3 minutes after the injection and lasted 30–40 minutes. In only 2 GP tested cells (4%), dopamine decreased the firing rate with a percentage decrease of 28% and in 14 GP neurons (28%) dopamine did not alter the firing rate (*p*>0.05, Fig. 1D, Table 1). In all GP tested neurons, local dopamine injection did not change the firing pattern as the coefficient of variation of the interspike intervals did not significantly change compared to before injection (*p>0*.*05*, Table 2). Analysis of the density histograms (Fig. 1AC) (16) showed similar results, e.g. the absence of significant changes of the firing pattern (*p*>0.05, Chi^2^ test).

**Table 1:** Firing rates of GP, STN and SNr neurons before and after dopamine or quinpirole injection into the GP.

	FR increase cells		FR decrease cells		Non responsive cells	
	*Before*	*After*	*Before*	*After*	*Before*	*After*
***GP neurons***						
Dopamine	18.64±1.62	27.03±2.44***	21.15±8.95	15.20±9.43*	22.63±2.07	22.63±2.12^ns^
Quinpirole	14.31±2.14	19.44±2.72**	20.22±3.31	10.87±1.56**	24.68±3.96	25.61±3.99^ns^
***STN neurons***						
Dopamine	4.72±0.60	6.66±0.96*	5.12±0.79	3.43±0.56*	6.39±0.72	6.22±0.60^ns^
Quinpirole	11.92±6.69	18.86±4.10*	11.36±2.51	7.23±1.43*	12.91±2.48	13.33±2.67^ns^
***SNr neurons***						
Dopamine	9.03±2.90	13.10±3.78*	9.41±1.24	5.73±1.03*	12.30±4.16	12.54±4.64^ns^
Quinpirole	21.82±6.00	32.12±7.40*	16.10±2.11	8.99±1.60*	12.91±2.48	13.33±2.67^ns^

FR: firing rate in spikes/sec; values are presented as the mean ± SEM. Statistical analysis using paired t-test was performed;

*: *p*<0.05,

**: *p*<0.01,

***: *p*<0.001 in comparison with before drug injection.

**Table 2:** Coefficient of variations of GP, STN and SNr neurons before and after dopamine or quinpirole injection into the GP.

	*GP neurons*		*STN neurons*		*SNr neurons*	
	*Before*	*After*	*Before*	*After*	*Before*	*After*
Dopamine	0.32±0.06	0.27±0.04^ns^	1.12±0.02	1.10±0.02^ns^	1.16±0.08	1.15±0.07^ns^
Quinpirole	0.34±0.06	0.36±0.06^ns^	1.12±0.03	1.12±0.03^ns^	1.18±0.06	1.16±0.05^ns^

Values are presented as the mean ± SEM. Statistical analysis using paired t-test was performed; ns: non significant difference in comparison with before drug injection.

**Fig 2:**
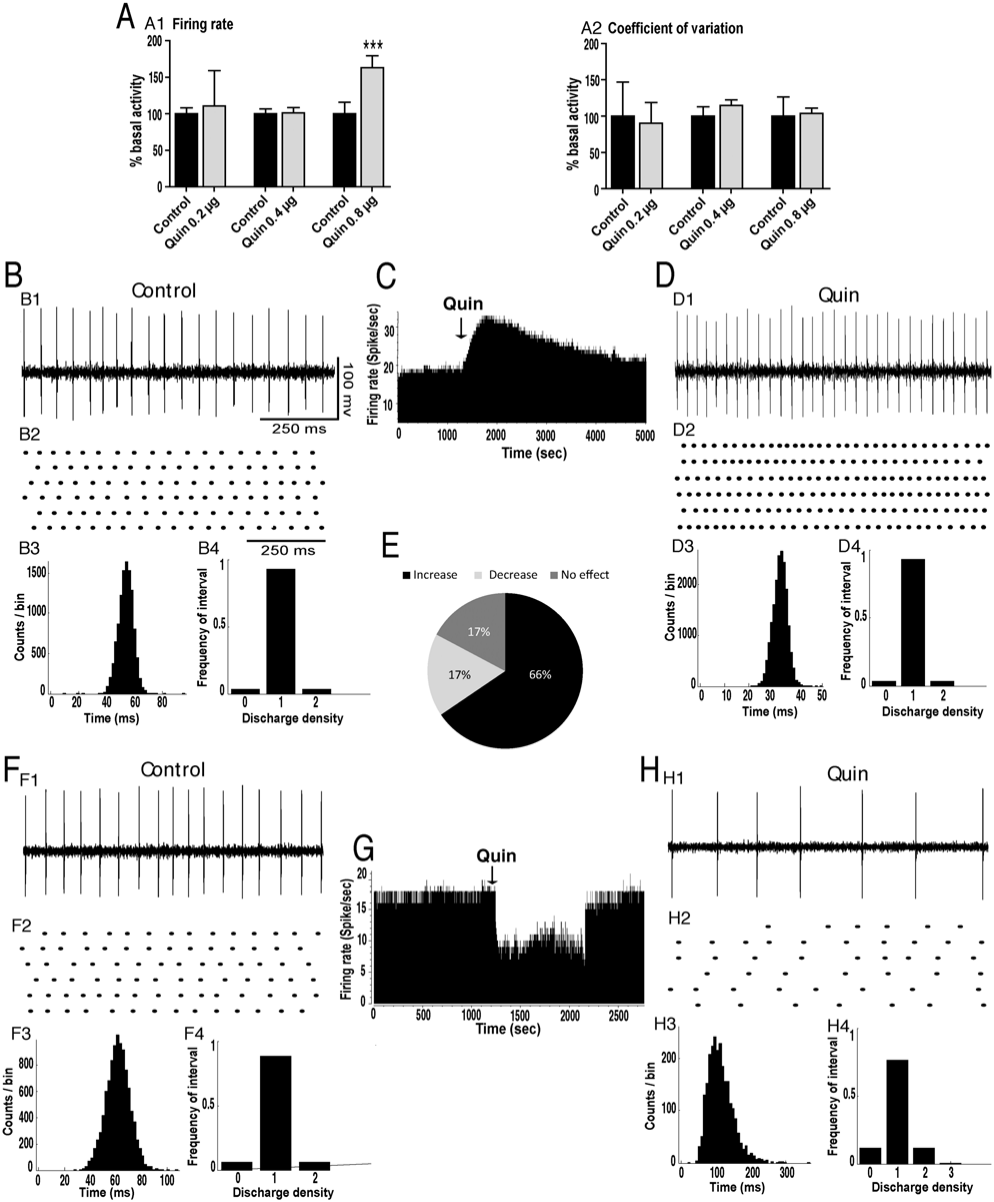
Intrapallidal microinjection of quinpirole predominantly increased the firing rate of GP neurons in a dose-dependent manner without changing the tonic firing pattern. (A) Histograms showing the dose response effects of quinpirole (Quin 0.2, 0.4 and 0.8 μg) on the firing rate (A1) and the coefficient of variation of the interspike intervals (A2) of GP neurons. ***p<0.001. (B-D) A representative example of GP neuron before (BC) and after (CD) microinjection of quinpirole (Quin) into the GP showing an increase of its firing rate with spike train (B1D1), firing rate histogram (C), raster display of random segments of recording (B2D2), insterspike interval histogram (B3D3) and density histogram (B4D4) of the same GP neuron. (E) Circular plot representing the percentage of GP neurons showing an increase, a decrease or no change of their firing rate after the local injection of quinpirole. (F-H) A representative example of GP neuron before (FG) and after (GH) microinjection of quinpirole (Quin) into the GP showing a decrease of its firing rate with spike train (F1H1), firing rate histogram (G), raster display of random segments of recording (F2H2), insterspike interval histogram (F3H3) and density histogram (F4H4) of the same GP neuron.

To test the hypothesis that the increased firing rate observed in GP cells after local microinjection of dopamine results from the activation of dopamine D2Rs, these receptors were blocked by the injection of sulpiride, a selective D2R antagonist. In all GP tested cells, sulpiride blocked the excitatory effect induced by dopamine (Fig. 1E). To confirm the importance of the D2Rs in the effects induced by dopamine, we tested the effect of intrapallidal injection of quinpirole, a selective D2R agonist. First, in a set of experiments, we investigated the dose response of quinpirole at the doses of 0.2, 0.4, and 0.8 μg. Local microinjection of quinpirole significantly affected the firing rate of GP neurons in a dose-dependent manner (F = 26.42, p<0.001, Fig. 2A). In contrast to the doses of 0.2 and 0.4 μg, which did not affect the firing rate (p>0.05 for the two doses), 0.8 μg significantly increased the firing rate of the majority of GP neurons (19 out of 29, 66%) with a percentage increase of 36% (*p*<0.01, paired *t*-test, Fig. 2A–E, Table 1). In 5 out of 29 GP tested cells (17%), quinpirole significantly decreased the firing rate (-46.24%, *p*<0.01, Fig. 2F–H, Table 1) and in 5 out of 29 GP tested neurons (17%) dopamine did not significantly alter the firing rate (*p*>0.05, Table 1). In all GP tested neurons, quinpirole did not change the firing pattern as the coefficient of variation of the interspike intervals did not significantly change after the injection of quinpirole compared to control conditions (*p*>0.05, Fig. 2, Table 2). Analysis of the density histograms showed similar results, e.g. the absence of significant changes in the firing pattern (*p*>0.05, Chi^2^ test).

### Effects of intrapallidal injection of dopamine and quinpirole on the spontaneous firing of STN neurons

**Fig 3:**
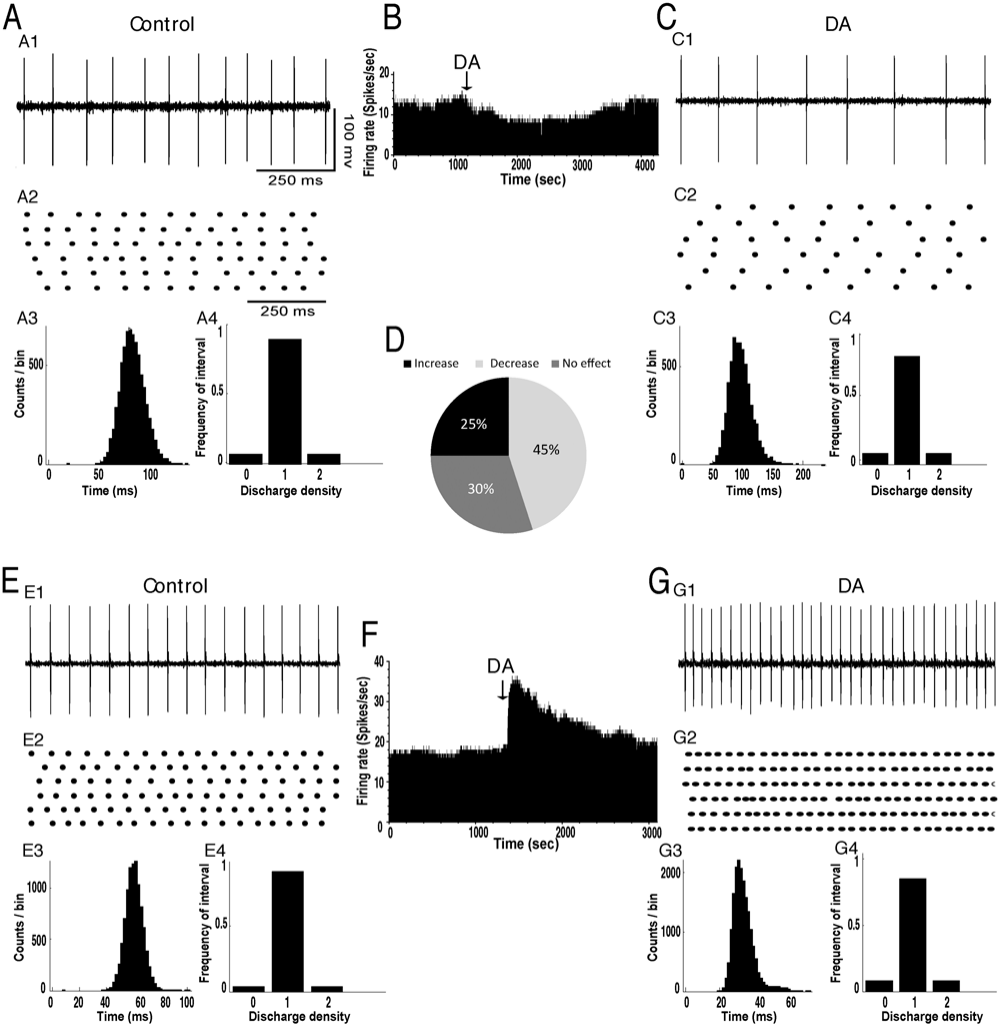
Intrapallidal microinjection of dopamine predominantly decreased the firing rate without changing the tonic firing pattern of STN neurons. (A-C) A representative example of STN neuron before (A) and after (C) microinjection of dopamine into the GP showing a decrease of its firing rate with spike train (A1C1), firing rate histogram (B) raster display of random segments of recording (A2C2), insterspike interval histogram (A3C3) and density histogram (A4C4) of the same STN neuron. (D) Circular plot representing the percentage of STN neurons showing an increase, a decrease or no change of their firing rate after the local injection of dopamine. (E-G) A representative example of STN neuron before (EF) and after (FG) microinjection of dopamine into the GP with spike train (E1G1), firing rate histogram (F), raster display of random segments of recording (E2G2), insterspike interval histogram (E3G3) and density histogram (E4G4) of the same STN neuron.

In basal conditions, the mean firing rate of STN neurons was 10.38±1.27 spikes/sec (n = 95 neurons in 27 rats) and most of the cells exhibited a tonic discharge pattern as shown by the interspike intervals and density histograms (Fig. 3A). Control microinjection of saline (0.9% NaCl) into the GP revealed no significant effects on the firing rate and pattern of neuronal activity in the STN (data not shown). However, dopamine injection into GP predominantly induced an inhibitory effect on STN neurons. It significantly decreased the firing rate of 9 out of 20 STN neurons (45%) with a percentage decrease of 33% (*p*<0.05, paired *t*-test, Fig. 3A–D, Table 1). This effect occurred within 2–3 minutes after the injection and lasted 30–40 minutes. In 5 out of 20 STN tested cells (25%), dopamine significantly increased the firing rate (p<0.05, Fig. 3E–G, Table 1) and in 6 out of 20 STN tested neurons (30%), dopamine did not significantly change the firing rate (*p>*0.05, Table 1). In all STN tested neurons, dopamine did not change the firing pattern as the coefficient of variation of the interspike intervals did not significantly change after dopamine injection into GP compared to control conditions (*p*>0.05, Fig. 3, Table 2). Analysis of the density histograms also showed the absence of changes of the firing pattern (*p*>0.05, Chi^2^ test).

**Fig 4:**
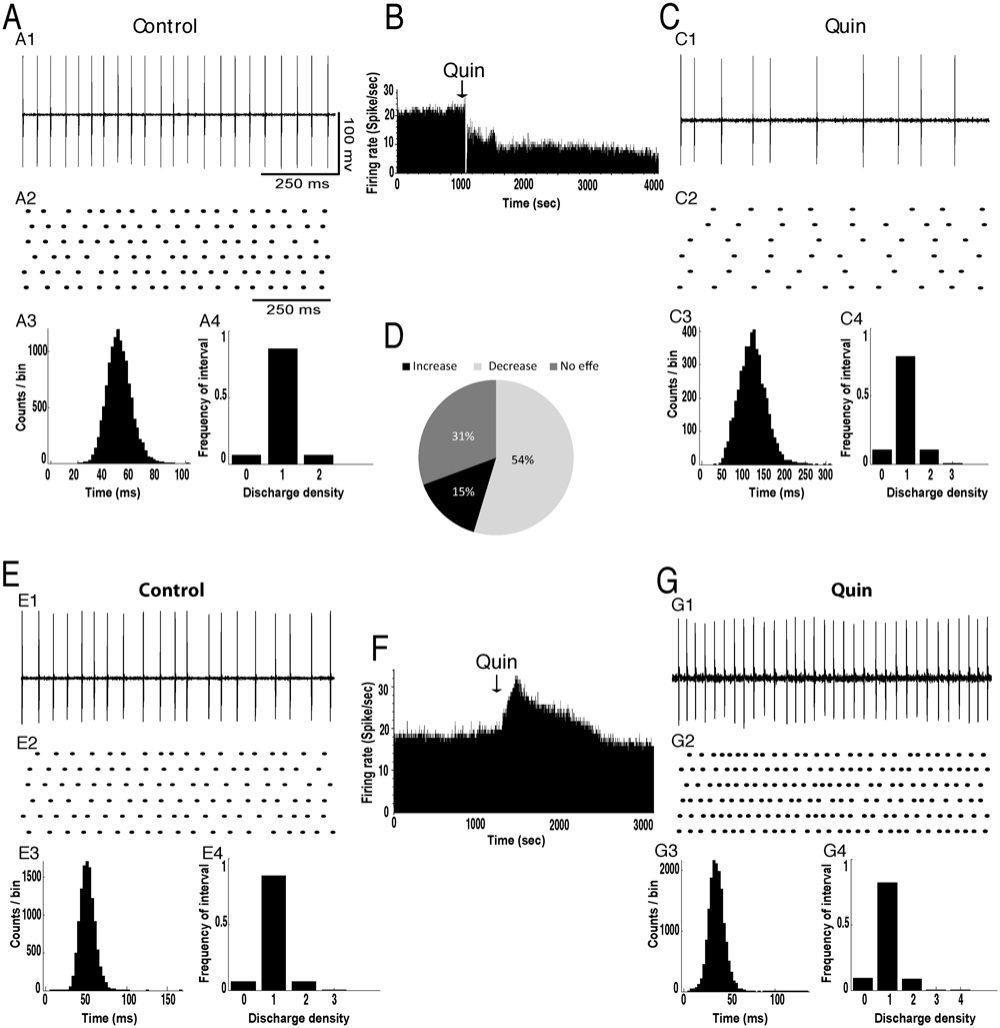
Intrapallidal microinjection of quinpirole predominantly decreased the firing rate without changing the tonic firing pattern of STN neurons. (A-C) A representative example of STN neuron before (A) and after (C) microinjection of quinpirole into the GP showing a decrease of its firing rate with spike train (A1C1), firing rate histogram (B), raster display of random segments of recording (A2C2), insterspike interval histogram (A3C3) and density histogram (A4C4) of the same STN neuron. (D) Circular plot representing the percentage of STN neurons showing an increase, a decrease or no change of their firing rate after the local injection of dopamine. (E-G) A representative example of STN neuron before (E) and after (G) microinjection of quinpirole into the GP showing an increase of its firing rate with spike train (E1G1), firing rate histogram (F), raster display of random segments of recording (E2G2), insterspike interval histogram (E3G3) and density histogram (E4G4) of the same STN neuron.

Intrapallidal injection of quinpirole into the GP significantly affected the firing rate of STN neurons. It decreased the firing rate of 41 out of 75 STN neurons (55%) with a percentage decrease of 36% (*p*<0.05, Fig. 4A–D, Table 1). In only 11 out of 75 STN tested cells (15%), quinpirole injection significantly increased the firing with a percentage increase of 58% (*p*<0.05, Fig. 4E–G, Table 1). In 23 out of 75 STN tested neurons (31%), quinpirole injection did not significantly change the firing rate (*p*>0.05, Fig. 4, Table 1). In all STN tested cells, quinpirole injection did not change the firing pattern as the coefficient of variation of the interspike intervals did not significantly change after the injection of dopamine compared to control conditions (*p*>0.05, Fig. 4, Table 2). Analysis of the density histograms also showed an absence of changes of the firing pattern (*p*>0.05, Chi2 test).

### Effects of intrapallidal injection of dopamine and quinpirole on the spontaneous firing of SNr neurons

**Fig 5:**
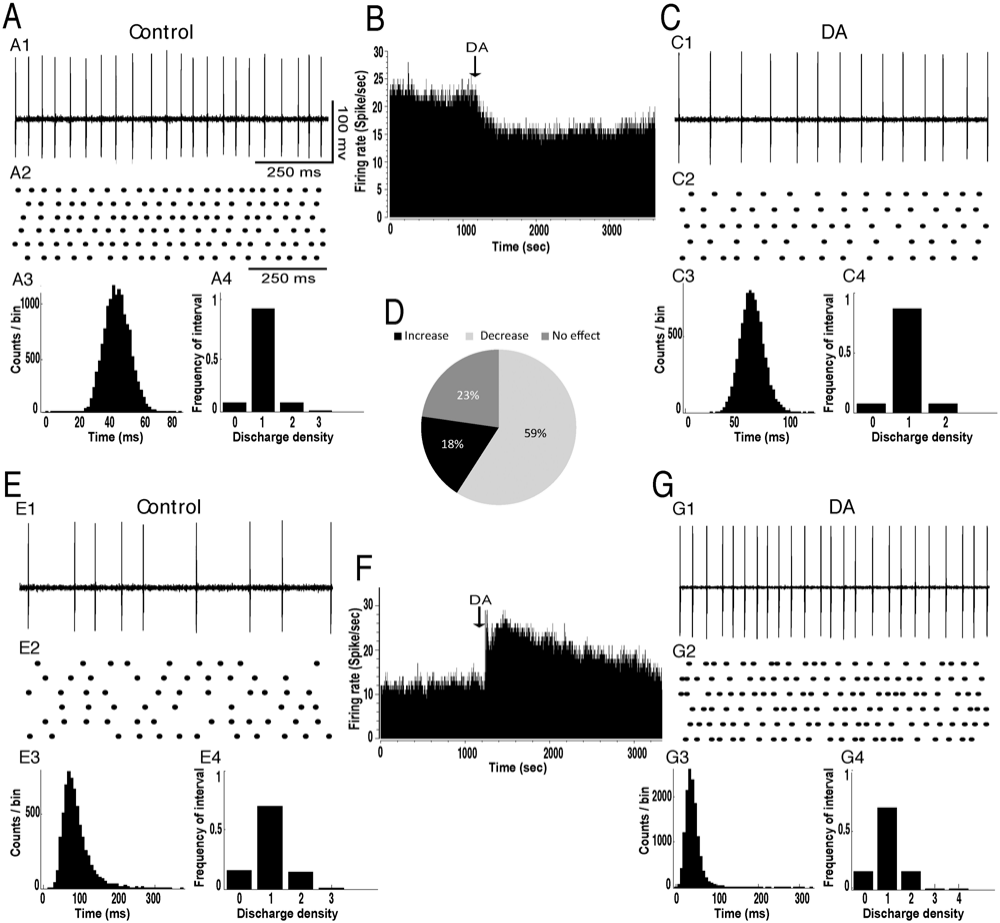
Intrapallidal microinjection of dopamine predominantly decreased the firing rate without changing the tonic firing pattern of SNr neurons. (A-C) A representative example of SNr neuron before (A) and after (C) microinjection of dopamine into the GP showing a decrease of its firing rate with spike train (A1C1), firing rate histogram (B), raster display of random segments of recording (A2C2), insterspike interval histogram (A3C3) and density histogram (A4C4) of the same SNr neuron. (D) Circular plot representing the percentage of SNr neurons showing an increase, a decrease or no change of their firing rate after the local injection of dopamine. (E-G) A representative example of SNr neuron before (E) and after (G) microinjection of dopamine into the GP showing an increase of its firing rate with spike train (D1F1), firing rate histogram (F), raster display (E2G2), insterspike interval histogram (E3G3) and density histogram (E4G4) of the same SNr neuron.

In basal conditions, the mean firing rate of SNr neurons was 15.52±1.37 spikes/sec (n = 107 neurons in 15 rats) and most of the cells exhibited a regular discharge pattern as shown by the coefficient of variation of the interspike intervals (Fig. 5A). Control microinjection of saline (0.9% NaCl) into the GP revealed no significant effects on the firing rate and pattern of neuronal activity in the SNr (data not shown). However, dopamine injection into GP predominantly induced an inhibitory effect on SNr neurons. It decreased the firing rate of 13 out of 22 SNr neurons (59%) with a percentage decrease of 39% (p<0.05, Fig. 5A–D, Table 1). This effect occurred within 2–3 minutes after the injection and lasted 30–40 minutes. In 5 out of 22 SNr tested cells (18%), dopamine significantly increased the firing rate with a percentage increase of 45% (p<0.05, Fig. 5E–G, Table 1) and in 5 out of 22 SNr tested neurons (23%) dopamine did not significantly change the firing rate (p>0.05, Table 1). In all SNr tested neurons, dopamine did not change the firing pattern as the coefficient of variation of the interspike intervals did not significantly change after dopamine injection into GP compared to control conditions (*p*>0.05, Fig. 5, Table 2). Analysis of the density histograms also showed the absence of changes of the firing pattern (p>0.05, Chi2 test).

**Fig 6:**
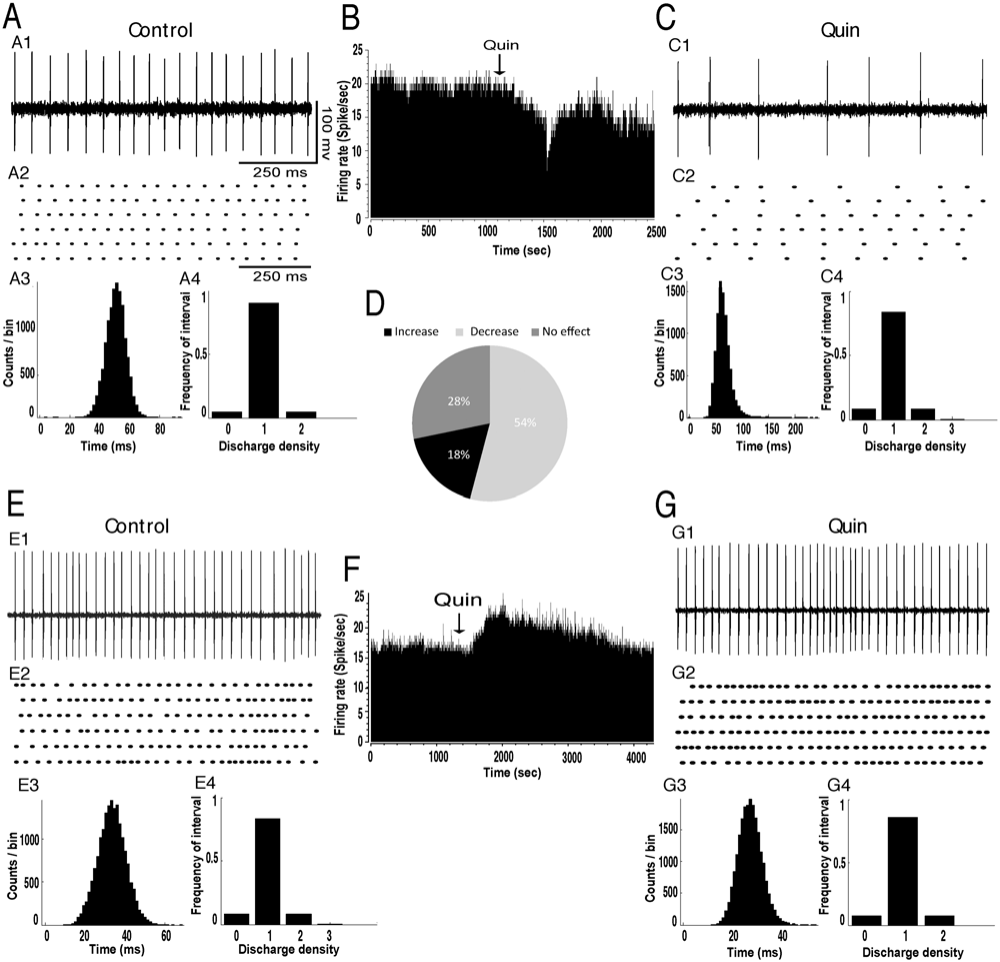
Intrapallidal microinjection of quinpirole predominantly decreased the firing rate without changing the tonic firing pattern of SNr neurons. (A-C) A representative example of SNr neuron before (A) and after (C) microinjection of quinpirole into the GP showing a decrease of its firing rate with spike train (A1C1), firing rate histogram (B), raster display of random segments of recording (A2C2), insterspike interval histogram (A3C3) and density histogram (A4C4) of the same SNr neuron. (D) Circular plot representing the percentage of SNr neurons showing an increase, a decrease or no change of their firing rate after the local injection of dopamine. (E-G) A representative example of SNr neuron before (E) and after (G) microinjection of quinpirole into the GP showing an increase of its firing rate with spike train (E1G1), firing rate histogram (F), raster display (E2G2), insterspike interval histogram (E3G3) and density histogram (E4G4) of the same SNr neuron.

Correspondingly, local microinjection of quinpirole into the GP significantly affected the firing rate of SNr neurons. Quinpirole significantly decreased the firing rate of 46 out of 85 SNr neurons (55%) with a percentage decrease of 44% (p<0.05, Fig. 6A–D, Table 1). In only 15 out of 85 SNr tested cells (18%), quinpirole injection significantly increased the firing rate with a percentage increase of 47% (p<0.05, Fig. 6E–G, Table 1) and in 24 out of 85 SNr tested neurons (28%), quinpirole injection did not significantly change the firing rate (p>0.05, Table 1). In all SNr tested cells, quinpirole injection did not change the firing pattern as the coefficient of variation of the interspike intervals did not significantly change after the injection of quinpirole compared to control conditions (*p*>0.05, Fig. 6, Table 2). Analysis of the density histograms also showed an absence of changes of the firing pattern (p>0.05, Chi2 test).

## Discussion

### Dopamine, through activation of D2Rs, modulates the firing rate but not the pattern of GP neurons

In the present study, we provide evidence that dopamine, through activation of D2Rs, exerts an excitatory effect on the majority of GP neurons *in vivo*. Thus, dopamine-induced firing rate increase was mimicked by the selective D2R agonist, quinpirole, and prevented by the selective D2R antagonist, sulpiride. However, neither dopamine nor quinpirole changed the discharge pattern, demonstrating that dopamine, through activation of D2Rs, modulates the firing rate but not the pattern of GP neurons under physiological conditions. The possibility that the absence of change of the firing pattern may be influenced by the use of urethane anesthesia cannot be completely rule out. However, this possibility may be minimized, as dopamine depletion in the rat, under the same conditions of anesthesia, has been shown to induce burst activity in GP neurons [19] and also in STN neurons [13,17,20,21]. Furthermore, similar burst activity has been reported in non-anesthetized MPTP-treated monkeys [22] and in patients with Parkinson’s disease [23,24]. Our results are consistent with previous studies showing that quinpirole increased the firing rate of GP neurons in the rat [11] and of GPe neurons in non-human primate [25]. Quinpirole also increased the expression of the immediate early gene c-fos, which is a marker of neuronal activity [26]. The firing rate increase, which represents the major effect of dopamine on GP neurons, may be explained by the action of this neurotransmitter on D2Rs located pre-synaptically on GABA striato-pallidal fibers [6]. Thus, dopamine, like quinpirole, reduces GABA release by activating D2Rs, resulting in a disinhibition of GP neurons. This is consistent with an early study, which showed that iontophoretic injection of dopamine or amphetamine reduced GABA transmission in the GP [27]. Accordingly, *in vitro* data demonstrated that dopamine, through activation of D2Rs on striato-pallidal terminals, exerts an inhibitory effect on GABA release in the rat GP [28,29]. These studies, together with ours, suggest that the striato-pallidal GABAergic inhibition is under the control of presynaptic D2Rs and that local depletion of dopamine may contribute to the changes in GP neuronal activity observed in animal models of Parkinson’s disease. Thus, intrapallidal injection of 6-hydroxydopamine-induced dopamine depletion in GP resulted in a decrease of the firing rate of GP neurons [12]. This supports the key role played by dopamine at extrastriatal sites, suggesting that dopaminergic drugs may play their anti-parkinsonian effects through activation of D2Rs located in GP in addition to their action in the striatum.

In addition to their localization on GABA fibers, D2Rs are also located presynaptically on glutamatergic afferents originating from the STN and the parafascicular nucleus of the thalamus [6]. Their activation has been suggested to reduce glutamatergic release in GP of *in vitro* slices [30]. This may explain why in some of our GP tested cells dopamine, like quinpirole, reduced their firing rate. The fact that this effect was observed in only a minority of GP neurons compared to those showing an increase in their firing rate, is consistent with the reduced number of D2Rs located on glutamate terminals compared to those located on GABA terminals [6]. In another population of GP tested cells, dopamine like quinpirole did not affect their firing activity. This can be due to the absence of dopamine receptors on afferents of these neurons. This result is consistent with data of a previous anatomical study showing that D2Rs were not found in all GP neurons but only in a population of approximately 40–50% [29].

We therefore postulate that dopamine acting at presynaptic D2Rs predominantly reduces GABA release at GABAergic terminals in GP. Presynaptic rather than postsynaptic dopaminergic modulation of GABAergic transmission in the GP is supported by the action of dopamine on miniature GABAergic transmission, which can be mimicked by the use of selective D2R agonists [28]. The firing rate increase of pallidal neurons caused by dopamine and its D2R agonist, quinpirole, would lead to decreased firing rate of their major basal ganglia efferent structures such as the STN and SNr.

### Dopamine, through activation of D2Rs in GP, modulates the GP-STN and GP-SNr neurotransmission by controlling the firing rate but not the pattern of STN and SNr neurons

The principal GABAergic input to the STN arises from the GP, which plays a key role in the control of firing activity of STN neurons. *In vitro* electrophysiology studies reported that spontaneous pallido-subthalamic activity influenced STN neuronal firing [31] and that electrical stimulation of GP afferents evoked IPSP or IPSC through activation of postsynaptic GABA_A_ receptors [32–34]. Here, we focused our study on the impact of dopaminergic modulation of GP-STN neurotransmission and we showed that dopamine, like quinpirole, when injected into the GP decreased the firing rate of most STN neurons. These results can be explained by the fact that dopamine, through activation of D2Rs, predominantly increased the firing rate of GP cells (present study), at the origin of GABA release in the STN, resulting in a reduction of the firing activity of majority of STN recorded neurons. This is the first study showing that DA in GP participates in the modulation of GP-STN neurotransmission and consequently controls STN neuronal firing. The inhibitory effect is mediated by GABA_A_Rs, as they are concentrated at GP-STN synapses and that GABA_A_R antagonists block spontaneous IPSCs [35]. Furthermore, we showed that DA, via D2Rs, increased the firing rate of a minority of STN neurons (25% for DA and 15% for quinpirole). This excitatory effect can be explained by the fact that dopamine, via D2Rs, reduces the firing rate of a small sub-population of GP cells inducing a decrease of GABA release in the STN, which in turn results in a disinhibition of STN neurons.

In the two populations of STN responsive neurons, the firing rate changes were not accompanied by a change in firing pattern. Together, our data show that DA participates in the modulation of the GP-STN pathway, contributing to the control of firing rate but not pattern of STN neurons. This is consistent with previous studies showing that the pattern of GP inhibitory input to the STN is crucial in determining whether STN neurons fire in a tonic or burst pattern [32], and that burst activity in GP neurons is necessary to generate sufficient hyperpolarization in STN neurons for rebound burst activity [36].

In addition to the control of GP-STN pathway, we show that dopamine in GP modulates the neuronal activity of the principal output structure of basal ganglia network in the rat, the SNr. We show that the responses of SNr neurons to pallidal microinjection of dopamine, or its D2R agonist, are similar to those of STN neurons (including decreases, increases and some neurons showing no change) with the same proportions. The changes observed in SNr neurons can be due to i) the activation of GABAergic neurons of GP projecting directly to the SNr or ii) to the deactivation of STN neurons projecting to the SNr as majority of STN neurons are inhibited by dopamine when injected in the GP or iii) to a combination of the two phenomena.

According to previous studies, it is likely that SNr cell responses to dopamine in GP are a consequence of the two phenomena. The first hypothesis is supported by anatomical tracing findings showing that individual GP neurons that project to the STN possess axon collaterals innervating the SNr (*for review*, Smith, Bevan [37], Deniau, Mailly [38]). Furthermore, in rat brain slices preparation, it has been shown that GP neurons have a significant impact on the discharge of SNr cells [38]. Indeed, GP stimulation evoked IPSPs of SNr neurons, which is strong enough to reset the firing of the neurons (38). The second hypothesis is supported by a previous electrophysiological study showing that the STN lesions induced an attenuation of changes in mean firing rate of SNr neurons in response to intrastriatal microinjection of apomorphine [39]. Based on these evidences, it is likely that SNr neuronal responses are due to changes in the level of activity of inhibitory (GP) and excitatory (STN) afferents and that both GP-SNr and GP-STN-SNr are important in the inhibitory response of SNr neurons to dopamine and quinpirole injection into GP.

In conclusion, our data are the first to show that dopamine, through activation of D2Rs located in the GP, plays a key role in modulating GP neuronal activity, which participates to the control of its two principal efferent projections, the STN and SNr. The predominant effect was an increase in the firing rate of GP neurons, resulting in the inhibition of GABA release from presynaptic terminals in the STN and SNr, leading to decreased activity of its neurons. In addition, the changes in STN neuronal activity may participate to the modulation of SNr neurons. Our data provide evidence that dopamine in GP controls the firing rate but not the pattern of GP neurons, which in turn control the firing rate, but not the pattern of STN and SNr neurons.
